# ERdj5 Is the ER Reductase that Catalyzes the Removal of Non-Native Disulfides and Correct Folding of the LDL Receptor

**DOI:** 10.1016/j.molcel.2013.05.014

**Published:** 2013-06-27

**Authors:** Ojore Benedict Valentine Oka, Marie Anne Pringle, Isabel Myriam Schopp, Ineke Braakman, Neil John Bulleid

**Affiliations:** 1 Institute of Molecular, Cellular and Systems Biology, College of Medical Veterinary and Life Sciences, Davidson Building, University of Glasgow, Glasgow G12 8QQ, UK; 2 Cellular Protein Chemistry, Faculty of Science, Utrecht University, 3584 CH Utrecht, the Netherlands

## Abstract

ERdj5 is a member of the protein disulfide isomerase family of proteins localized to the endoplasmic reticulum (ER) of mammalian cells. To date, only a limited number of substrates for ERdj5 are known. Here we identify a number of endogenous substrates that form mixed disulfides with ERdj5, greatly expanding its client repertoire. ERdj5 previously had been thought to exclusively reduce disulfides in proteins destined for dislocation to the cytosol for degradation. However, we demonstrate here that for one of the identified substrates, the low-density lipoprotein receptor (LDLR), ERdj5 is required not for degradation, but rather for efficient folding. Our results demonstrate that the crucial role of ERdj5 is to reduce non-native disulfides formed during productive folding and that this requirement is dependent on its interaction with BiP. Hence, ERdj5 acts as the ER reductase, both preparing misfolded proteins for degradation and catalyzing the folding of proteins that form obligatory non-native disulfides.

## Highlights

• Several endogenous substrates for ERdj5 have been identified
• ERdj5 is required for efficient folding and secretion, but not degradation, of LDLR
• Knockdown of ERdj5 prevents the removal of non-native disulfides
• ERdj5 needs to interact with BiP to carry out its function during protein folding

## Introduction

ERdj5 is an endoplasmic reticulum (ER)-localized oxidoreductase that contains a J domain as well as six thioredoxin domains, four of which are responsible for its disulfide exchange activity (Cunnea et al., 2003; Hosoda et al., 2003). Our existing knowledge of the function of ERdj5 indicates a role in ER-associated degradation (ERAD) (Dong et al., 2008; Ushioda et al., 2008). It has been shown to interact with components of the degradation pathway (Christianson et al., 2012; Hagiwara et al., 2011), in particular EDEM1, which recognizes proteins destined for degradation and targets them to the ER membrane for subsequent dislocation into the cytosol (Cormier et al., 2009; Groisman et al., 2011). Overexpression of wild-type (WT), but not an active-site mutant of ERdj5, accelerates the degradation of model proteins such as the null Hong Kong (NHK) variant of α1 antitrypsin and the J chain of immunoglobulin M (IgM) (Ushioda et al., 2008). Both of these proteins form intermolecular disulfides that need to be reduced for the protein to be degraded efficiently. The accelerated degradation of these substrates was prevented by the inclusion of a drug that inhibits trimming of the oligosaccharide side chain. As this trimming event is required for glycoproteins to be recognized by the machinery for ERAD (Wilson et al., 2000), this result indicates that it is the physical association of ERdj5 with EDEM1 that targets the substrate for disulfide reduction. Hence, ERdj5 is thought to catalyze the reduction of disulfides in substrates already targeted for degradation, an activity that prepares the protein for subsequent passage through the ER membrane.

In addition to its interaction with EDEM1, ERdj5 also binds to the ER-localized Hsp70 homolog BiP (Cunnea et al., 2003; Ushioda et al., 2008). Binding is ATP dependent and requires the J domain, as its removal prevents BiP binding. The sequence HPD within the J domain is known to mediate binding to Hsp70. As expected, BiP binding was prevented when this sequence in ERdj5 was mutated to QPD. Blocking the interaction between ERdj5 and BiP prevented the accelerated degradation of model substrates caused by overexpression of ERdj5, indicating that this oxidoreductase functions during degradation by binding to BiP.

The ER oxidoreductases are a large family of disulfide exchange proteins characterized by the presence of at least one catalytically active thioredoxin domain containing a CXXC motif (Ellgaard and Ruddock, 2005). ERdj5 is distinct in this family not only for the presence of a J domain but also due to the reduction potential of its active-site cysteines. While there is some variability of reduction potential between the four active-site disulfides in ERdj5, they are generally more stable than other protein disulfide isomerase (PDI) family members and are more likely to accept electrons and act as reducing agents (Ushioda et al., 2008). Studies with the purified protein have shown that, although it is able to reduce disulfides in substrates, it does not catalyze disulfide formation or the isomerization of non-native disulfides (Ushioda et al., 2008). Hence, the biophysical properties and the in vitro analysis of ERdj5 are consistent with its role as a reductase in the cell.

To date, the number of known protein substrates for ERdj5 is quite limited and is focused on those that require disulfide reduction prior to degradation. One approach that has been used in the past to identify novel substrates for PDI family members involves the expression of mutant enzymes in which the second cysteine in the active site has been mutated to alanine (Jessop et al., 2009a). Such a CXXA active site is unable to resolve any disulfides formed between enzyme and substrate, thereby allowing the isolation of disulfide-linked complexes. The isolated substrate can then be identified by mass spectrometry. Here we have carried out such an approach with ERdj5, thereby greatly expanding its potential substrate repertoire. One particular isolated substrate, the low-density lipoprotein receptor (LDLR), is known to form non-native disulfides during its folding pathway (Jansens et al., 2002). We show here that rather than being involved in the degradation of the LDLR, ERdj5 is in fact required for its efficient folding and secretion. Such a role for ERdj5 was unexpected and reveals the versatility of the PDI family in their biological functions.

## Results

### Increasing the Repertoire of Known Substrates for ERdj5

**Figure 1:**
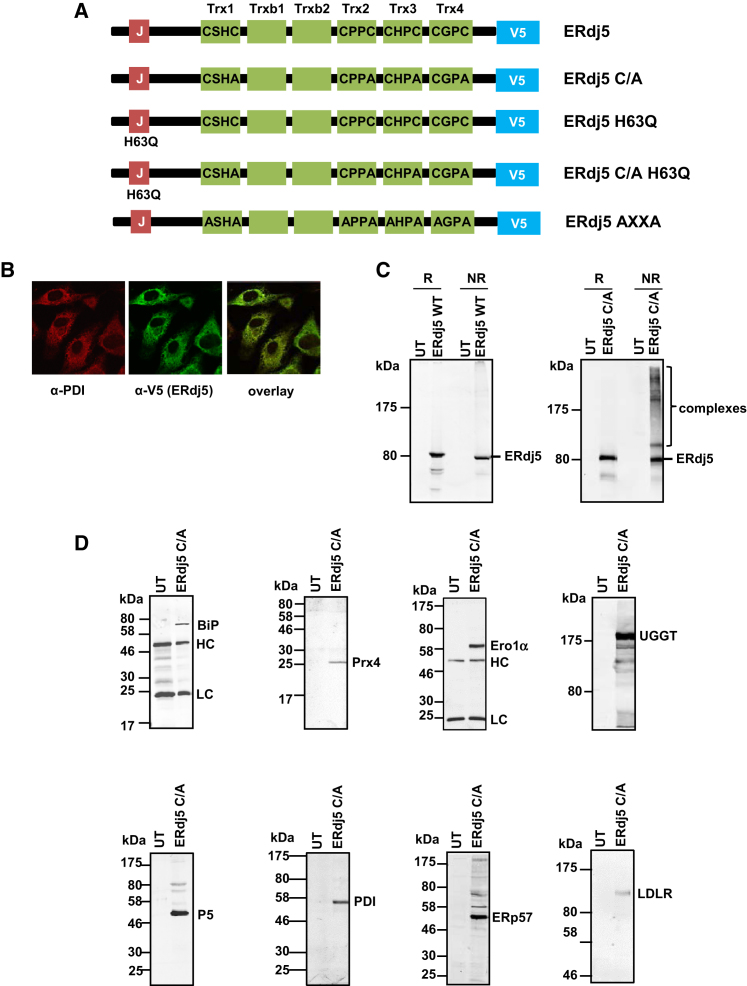
ERdj5 Substrate-Trapping Mutant Forms Mixed Disulfides with Proteins Entering the Secretory Pathway (A) Schematic of the various ERdj5 constructs used in this study depicting the domain organization of ERdj5 engineered with a C-terminal V5 tag but retaining a C-terminal KDEL sequence. Trx1–Trx4 are the thioredoxin domains with active sites as indicated, whereas Trxb1 and Trxb2 are thioredoxin domains without active sites. (B) Confocal immunofluorescence microscopy of HT1080 cells stably expressing ERdj5 C/A. Cells were fixed and stained with antibodies to the V5 epitope tag (green) and to an ER-localized protein PDI (red). (C) Cell lysates from HT1080 cells, either untransfected (UT), transiently expressing ERdj5 WT, or stably expressing ERdj5 C/A, separated under reducing (R) or nonreducing (NR) conditions were immunoblotted using the V5 antibody to detect the exogenously expressed ERdj5. (D) ERdj5 C/A was immunoisolated with the V5 antibody from cell lysates of HT1080 cells either untransfected (UT) or stably expressing ERdj5 C/A. Immunoisolates were analyzed by immunoblotting with antibodies to endogenous ER proteins and LDLR as indicated. HC and LC (anti-BiP and anti-Ero1 blots) indicate immunoglobulin heavy and light chains, respectively.

To identify the endogenous substrates of ERdj5, we created a stable cell line expressing a version of the enzyme that contained a CXXC-to-CXXA mutation at each of the four active sites (ERdj5 C/A: Figure 1A). Such active-site mutants of the thioredoxin family of proteins have been used previously to trap substrates in covalent complexes with the enzyme (Jessop et al., 2007, 2009b; Zito et al., 2010). We also appended a V5 epitope tag at the C terminus prior to the KDEL retention sequence to allow the identification and immunoisolation of the exogenously expressed protein. We confirmed that the protein was expressed and localized to the ER by immunofluorescent microscopy (Figure 1B). When the cell lysates were separated by nonreducing SDS-PAGE, several V5-reactive high-molecular-weight complexes were identified, that were lost when the samples were separated under reducing conditions and were not present when the wild-type protein was expressed (Figure 1C). This result demonstrates the presence of mixed disulfides between ERdj5 C/A and endogenous proteins. To identify these proteins, we immunoisolated V5-tagged ERdj5 and eluted disulfide-bonded partners with the reducing agent dithiothreitol (DTT). The eluted proteins were then digested with trypsin and the released peptides identified by mass spectrometry (Table 1). The formation of disulfide-linked complexes with several of the proteins identified was confirmed by their immunoisolation with the V5 antibody and detection by western blot (Figure 1D). The interactions were specific to exogenously expressed ERdj5, as none of the identified substrates were immunoisolated from untransfected cells.

**Figure 2:**
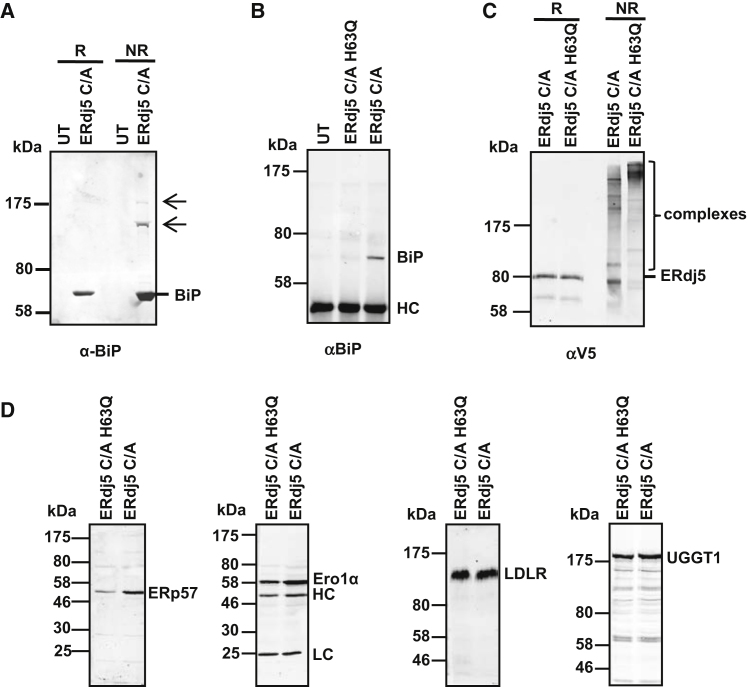
Preventing BiP Interaction with ERdj5 Does Not Prevent Mixed Disulfide Formation (A) Postnuclear lysates from untransfected HT1080 cells (UT) and ERdj5 C/A overexpression cells were immunoisolated with the V5 antibody. The immunoprecipitates were separated under reducing (R) or nonreducing (NR) conditions followed by rabbit anti-BiP western blot. Mixed disulfides are indicated with arrows. (B) ERdj5 was immunoisolated with the V5 antibody from cell lysates of untransfected HT1080 cells (UT), ERdj5 C/A-overexpressing HT1080 cells, and ERdj5 C/A H63Q-overexpressing HT1080 cells. Immunoisolated material was immunoblotted using a mouse anti-BiP antibody. HC indicates immunoglobulin heavy chains. (C) Cell lysates from ERdj5 C/A- and ERdj5 C/A H63Q-overexpressing cells were separated under reducing (R) or nonreducing (NR) conditions and immunoblotted using the V5 antibody. (D) ERdj5 was immunoisolated from cell lysates of HT1080 cells stably expressing either ERdj5 C/A or ERdj5 C/A H63Q. Immunoisolates were analyzed by immunoblotting with antibodies to ERdj5 client proteins as indicated. HC and LC (anti-Ero1 blot) indicate immunoglobulin heavy and light chains, respectively.

The identified proteins can be categorized into three groups: those that are resident to the ER, those that are soluble and secreted, and those that are integral membrane proteins localized to parts of the secretory pathway distinct from the ER. The ER resident proteins include several other PDI family members as well as PrxIV, Ero1, BiP, Grp94, and the formylglycine-generating enzyme. The formation of mixed disulfides between PDI family members has been seen previously (Jessop et al., 2009b) and may reflect some disulfide exchange reactions occurring between these proteins. PrxIV, Ero1, and the formylglycine-generating enzyme all form disulfides during their reaction cycles, so may require ERdj5 to break these disulfides (Bulleid, 2012; Dierks et al., 2005). An interaction with BiP was expected, as ERdj5 has been shown to associate with this protein (Cunnea et al., 2003). The fact that the interaction was released upon reduction could indicate a direct covalent link between BiP and ERdj5, a redox dependence of the ERdj5-BiP interaction, or an interaction between BiP and a substrate that itself forms a covalent link to ERdj5. Interestingly, we found that ERdj5 C/A formed a mixture of noncovalent and covalent interactions with BiP (Figure 2A), suggesting a role for this enzyme in the reduction of a disulfide found within BiP (Wei et al., 2012). All of the soluble and membrane-integrated proteins identified contain several cysteine residues or known disulfides, so it is likely that ERdj5 is involved in either their biosynthesis or degradation.

Since ERdj5 is known to interact with BiP via its J domain, we determined the consequence of preventing this association by mutating the J domain sequence HPD to QPD. We created a stable cell line expressing the substrate-trapping mutant of ERdj5 containing the H63Q mutation. The amount of BiP associating with ERdj5 was greatly diminished, as evidenced by the virtual absence of BiP in the V5-isolated material from the ERdj5 C/A H63Q compared to the ERdj5 C/A cell line (Figure 2B). However, mixed disulfide complexes were still present in this cell line, though their pattern of mobility was distinctly different from the complexes seen in the ERdj5 C/A cell line (Figure 2C). Such a shift in mobility of the mixed disulfide complexes could reflect a difference in the types of proteins forming mixed disulfides with ERdj5 or may be a result of different multicomponent complexes forming when ERdj5 is prevented from interacting with BiP via its J domain.

Identification of the V5-immunoisolated proteins that were eluted with DTT revealed that most of the previously identified proteins also formed mixed disulfides with ERdj5 C/A H63Q (Table 1). Some of the interacting partners of ERdj5 C/A H63Q also were confirmed by carrying out a western blot following immunoisolation of complexes with the V5 antibody (Figure 2D). Surprisingly, BiP was identified in the eluted proteins by mass spectrometry, even though the J domain mutation should block noncovalent interactions, and BiP was barely detected by western blotting in the V5-immunoisolated proteins (Figure 2B). This result reflects the increased sensitivity of mass spectrometry detection over immunodetection by western blotting. The ability of ERdj5 C/A H63Q to form mixed disulfides with endogenous proteins would suggest that ERdj5 can function as a reductase even in the absence of a J domain-mediated interaction with BiP.

### Characterization of the ERdj5 Interaction with the LDL Receptor

**Figure 3:**
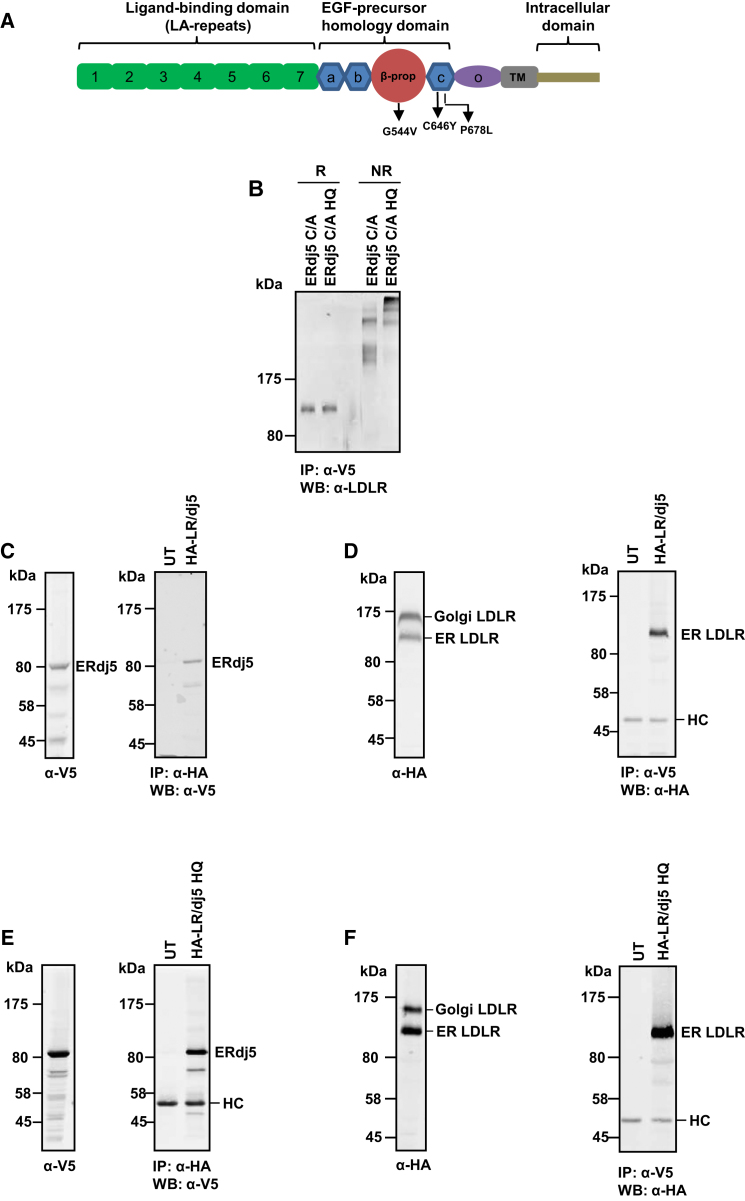
ERdj5 Forms Mixed Disulfide Complexes and Noncovalent Interactions with LDLR (A) Schematic of the domain organization of the LDLR, with the positions of the class 2 mutants used in this study indicated. (B) ERdj5 C/A or ERdj5 C/A H63Q were immunoisolated with the V5 antibody from cell lysates, separated under reducing (R) or nonreducing (NR) conditions, and immunoblotted for coisolated endogenous LDLR. (C) Cell lysate from HT1080 cells transiently transfected with HA-tagged LDLR (HA-LR) and ERdj5 (dj5) was immunoblotted with the V5 antibody prior to (left panel) or following (right panel) immunoisolation with the HA antibody. (D) Cell lysate from HT1080 cells coexpressing HA-LDLR and ERdj5 was immunoblotted with the HA antibody prior to (left panel) or following (right panel) immunoisolation with the V5 antibody. (E) Same as in (C), except HT1080 cells were cotransfected with HA-LDLR and ERdj5 H63Q. (F) Same as in (D), except HT1080 cells were cotransfected with HA-LDLR and ERdj5 H63Q.

As the folding of the LDLR has been studied extensively (Gent and Braakman, 2004; Pena et al., 2010), we decided to focus on this protein to characterize the role of ERdj5 during protein folding and secretion. The ectodomain of LDLR is composed of three regions (Figure 3A): an amino-terminal region containing seven ligand-binding repeats, an epidermal growth factor (EGF) precursor homology, and an *O*-linked glycosylated region. The multiple domains within LDLR previously have been shown to fold cooperatively with the formation of intra- or interdomain, non-native disulfides that need to be resolved to allow folding to proceed (Jansens et al., 2002). The identification of mixed disulfides between the LDLR and ERdj5 suggests a role for this enzyme in reduction of disulfides, either as a prerequisite for correct folding or during degradation.

To determine the extent of mixed disulfide formation between ERdj5 and the LDLR, we isolated V5-tagged ERdj5 from cell lines stably expressing either the C/A or the C/A-H63Q mutant. We then carried out western blots of the immunoisolate separated under reducing or nonreducing conditions (Figure 3B). All of the endogenous LDLR immunoisolated with ERdj5 was present as a mixture of disulfide-stabilized complexes. A different pattern of mixed disulfides was observed with the H63Q mutant of ERdj5 C/A, indicating that a lack of BiP interaction leads to a change in the type of mixed disulfides formed. Taken together, these results demonstrate the ability of ERdj5 to act as a reductase for LDLR and show that although the J domain mutant prevents BiP association, it does not prevent ERdj5 C/A from forming a mixed disulfide complex with its substrate.

Having established that a substrate-trapping mutant of ERdj5 interacts with LDLR, we then determined whether wild-type ERdj5 also interacts with LDLR. For these experiments, we coexpressed V5-tagged ERdj5 with HA-tagged LDLR and then carried out either a V5 or HA immunoisolation followed by an HA or V5 western blot of the immunoisolate (Figures 3C and 3D). The reciprocal partner was immunoisolated in both cases, demonstrating an interaction between these two proteins. LDLR migrates as two species when separated by SDS-PAGE: an ER form and a slower-migrating, Golgi-processed *O*-glycosylated form (Jansens et al., 2002) (Figure 3D, left panel). Only the ER form of the LDLR was immunoisolated with ERdj5, indicating that the complex dissociates prior to transport to the Golgi apparatus. When LDLR was coexpressed with the H63Q mutant of ERdj5, a similar association between these two proteins was observed (Figures 3E and 3F). These results highlight the physical interaction of ERdj5 with ER-localized LDLR and show that this interaction is not dependent on the binding of BiP to the J-domain of ERdj5. A comparison of the ratio of ER and Golgi forms of LDLR coexpressed with wild-type ERdj5 or the H63Q mutant reveals that there is a relative abundance of the ER form when LDLR is expressed with the H63Q mutant (compare Figures 3D and 3F). In addition, we consistently saw more of LDLR coimmunoisolated when ERdj5 H63Q was coexpressed. These results suggest a prolonged interaction of LDLR with the ERdj5 H63Q mutant, resulting in retention of LDLR in the ER.

### ERdj5 and the Degradation of LDLR

**Figure 4:**
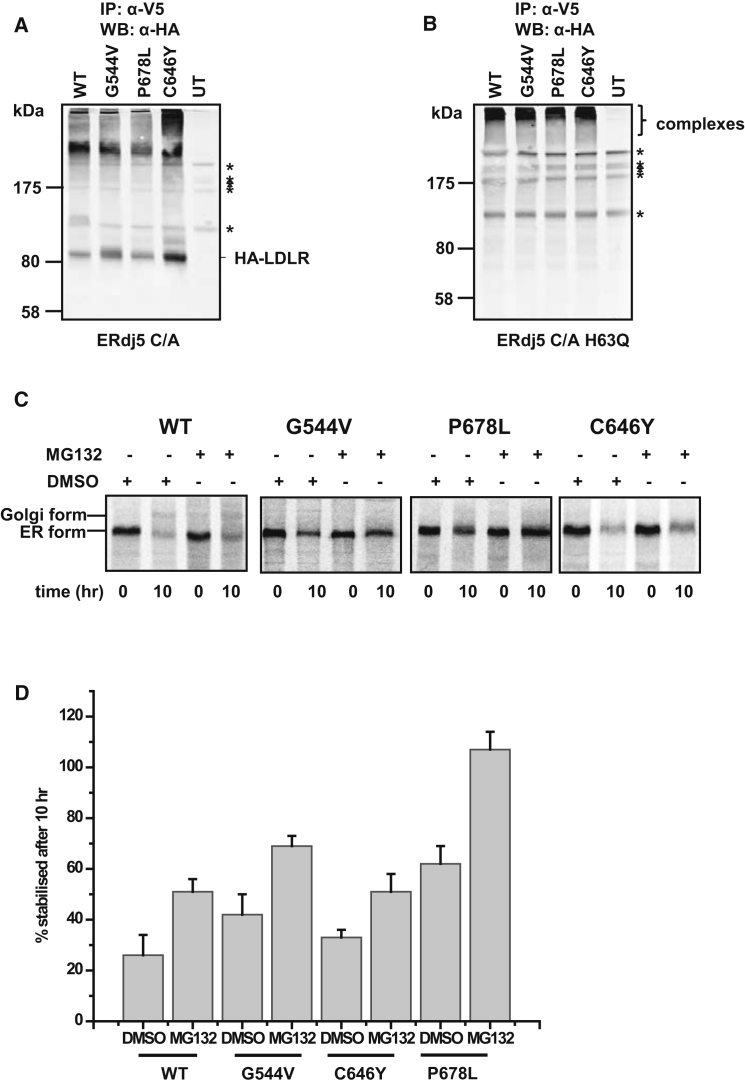
LDLR Class 2 Mutants Interact with ERdj5 and Are Substrates for ERAD (A and B) ERdj5 C/A- (A) or ERdj5 C/A H63Q (B)-overexpressing cells were transfected with wild-type HA-LDLR or three class 2 mutants, G544V, P678L, or C646Y. ERdj5 was immunoisolated from individual transfections with the V5-antibody; samples were separated under nonreducing conditions, and any coisolated LDLR was detected by immunoblotting with the HA antibody. (C and D) HT1080 cells transfected with HA-tagged WT or mutant LDLR were radiolabelled for 30 min and chased for 0 and 10 hr in the presence of DMSO (control) or the proteasome inhibitors MG132. The remaining radiolabelled LDLR was immunoisolated from cell lysates and analyzed under reducing conditions (C). LDLR was detected by phosphorimage analysis, and the percent of HA-LDLR that was stabilized was quantified and is shown in (D). Error bars represent ±SD for at least three independent experiments. Asterisks depict non-LDLR proteins, as they are also present in the untransfected controls.

There are two potential roles that the reductase activity of ERdj5 might play in the maturation of LDLR: either reducing disulfides prior to degradation or reducing non-native disulfides during productive folding. To investigate the role of ERdj5 in degradation, we determined the contribution of ERAD to the turnover of ER-localized LDLR. Previous studies have demonstrated that wild-type LDLR is not subjected to ERAD; however, some of the LDLR class 2 mutants that misfold in the ER are degraded via this pathway (Li et al., 2004). Three such mutants (G544V, C646Y, and P678L) were first expressed in the ERdj5 C/A cell line to see if they also formed mixed disulfides (Figure 4). Each construct was tagged with HA so that we could distinguish the exogenously expressed protein from the endogenous LDLR. The mutants formed similar patterns of mixed disulfides with ERdj5 C/A (Figure 4A). Some of the LDLR immunoisolated with the V5-tagged ERdj5 C/A was not present as a mixed disulfide, indicating that it interacted noncovalently. This result contrasts the situation with endogenous LDLR (Figure 3B), likely reflecting the higher level of expression of LDLR following transient transfection. When the H63Q mutant of ERdj5 C/A was coexpressed with LDLR, only mixed disulfide complexes were isolated (Figure 4B). This result highlights the difference in the mixed disulfide species formed between LDLR and either ERdj5 C/A or ERdj5 C/A H63Q and suggests that, in the absence of BiP binding, LDLR forms more prolonged mixed disulfides with ERdj5.

To evaluate the role of ERAD in the degradation of both WT and the class 2 mutants of LDLR, we pulse-labeled and incubated cells for 10 hr in the presence or absence of the proteasome inhibitor MG132. We immunoisolated any radiolabelled LDLR remaining in order to determine the level of degradation or stabilization of ER-localized LDLR (Figures 4C and 4D). All three LDLR mutants were stabilized following treatment with MG132, confirming previous work (Li et al., 2004) demonstrating that they are subject to degradation by ERAD. Our result with the wild-type protein contrasts this previous work, as it does suggest some stabilization of the protein in the presence of the proteasome inhibitor.

**Figure 5:**
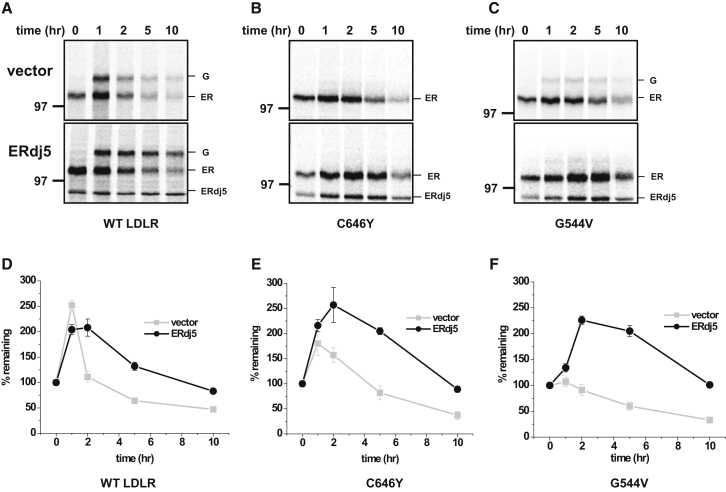
ERdj5 Promotes ER Retention, but Not Degradation, of LDLR Class 2 Mutants (A–C) HT1080 cells were cotransfected with either wild-type LDLR (A) or LDLR class 2 mutants C646Y (B) or G544V (C) and either an empty vector (top panel) or ERdj5 (bottom panel). Cells were pulse labeled for 30 min and chased for the indicated times. HA-LDLR was immunoisolated from the lysates with the HA antibody and analyzed under reducing conditions. (D) The ER and secreted forms of the WT HA-LDLR were quantified, and the percent of the total LDLR remaining was plotted versus the indicated chase times. (E and F) Percent of the ER form remaining for the class 2 mutants C646Y and G544V were quantified in (E) and (F), respectively. Error bars represent ±SD for at least three independent experiments.

To determine if ERdj5 plays a role in the degradation of the class 2 mutants, we took advantage of the previously described observation that overexpression of ERdj5 leads to an accelerated degradation of its substrates (Ushioda et al., 2008). We carried out a pulse-chase analysis to follow the transport and degradation of LDLR over a 10 hr period. No cycloheximide was added to inhibit translation, so there was an initial increase in radiolabelled protein, due to completion of synthesis of radiolabelled chains, followed by a decrease in signal. For wild-type LDLR, transport of the protein from the ER to the Golgi occurred within the first hr of the chase, followed by a steady decrease in the total signal over 10 hr (Figure 5A). Such a decrease likely reflects turnover following endocytosis and transport to the lysosome (Brown and Goldstein, 1975). In contrast, all of the C646Y, and most of the G544V, mutant was retained in the ER and was not transported to the Golgi (Figures 5B and 5C). Coexpression of ERdj5 had no effect on the transport of wild-type and did not lead to an acceleration of degradation of either mutant or wild-type LDLR (Figures 5D–5F). In fact, coexpression of ERdj5 actually led to a stabilization of the ER form of both LDLR mutants and increased the retention of the G544V mutant in the ER (Figure 5C, compare amounts of the Golgi form in upper and lower panels). These results suggest that ERdj5 is not involved in the degradation of misfolded LDLR, despite its association and ability to catalyze disulfide reduction. However, lack of an accelerated degradation of the LDLR mutants when ERdj5 is overexpressed does not rule out a role of this protein in their degradation; rather, it could mean that disulfide reduction is not a rate-limiting step in the degradation of these mutants.

### ERdj5 and Folding of the LDLR

**Figure 6:**
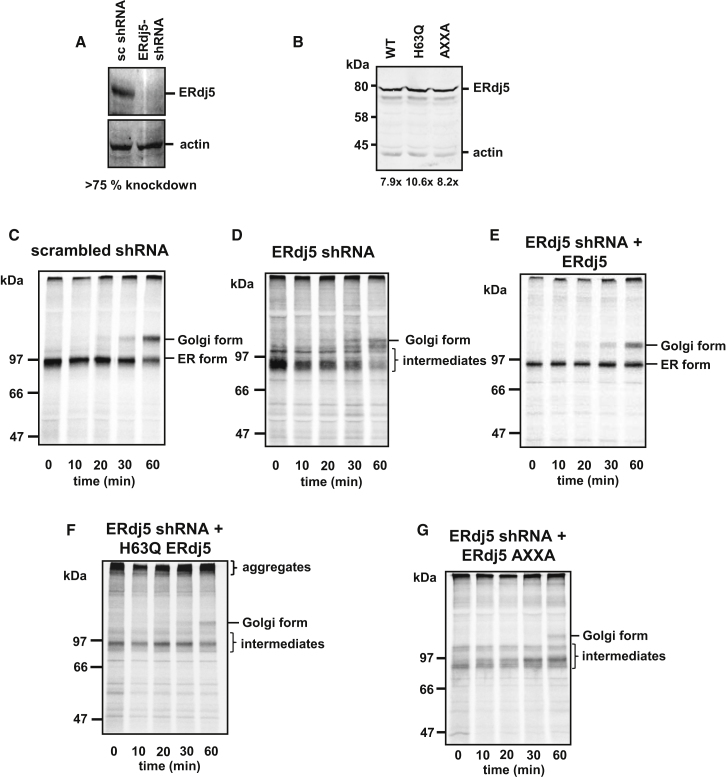
shRNA Knockdown of Endogenous ERdj5 Compromises Native Disulfide Bond Formation and Secretion of LDLR (A) HT1080 cells were treated with either ERdj5-specific or scrambled shRNA as indicated, and the level of ERdj5 remaining after 5 days was quantified using actin as a loading control. (B) Comparison of the level of ERdj5 in ERdj5 knockdown cells transfected with various ERdj5 constructs as indicated. Level of expression compared to nondepleted cells is as indicated. (C) HT1080 cells were treated with scrambled shRNA for 5 days and then cotransfected with HA-LDLR and empty vector. After a further 24 hr, cells were pulse labeled for 30 min and chased for the indicated times. Radiolabelled LDLR was immunoisolated from the cell lysate with the HA antibody and analyzed under nonreducing conditions. (D–G) HT1080 cells were treated with shRNA directed against ERdj5 then cotransfected with HA-LDLR and either empty vector (D), ERdj5 (E), ERdj5 H63Q (F), or ERdj5 AXXA mutant (G). Cells were pulse labeled for 30 min, and radiolabelled LDLR was isolated from the cell lysate and analyzed as in (C). The ER and Golgi forms are indicated as well as disulfide-bonded intermediates.

The lack of an accelerated degradation of LDLR by overexpression of ERdj5 suggested that the role of ERdj5 may be to assist correct folding. To test this possibility, cells were transfected with either a control small hairpin RNA (shRNA) or ERdj5 shRNA that resulted in a depletion of endogenous ERdj5 of >75% (Figure 6A). Subsequently, control and ERdj5-depleted cells were transfected with HA-LDLR. Folding and trafficking of HA-LDLR was followed by pulse-chase analysis. In the control cells, a diffuse band for immunoisolated LDLR can be seen immediately after the pulse (Figure 6C). Such a banding pattern is indicative of an ensemble of disulfide-bonded species being present at early time points, most of which are non-native disulfides (Jansens et al., 2002). After 10 min into the chase, a more distinct band is seen, with the appearance of the Golgi form of the protein becoming visible after 20 min. This time course contrasted sharply with that seen in ERdj5-depleted cells (Figure 6D). Here, the diffuse band seen at the start of the chase remained throughout the time course with only a small amount of protein being transported to the Golgi even after 60 min of chase. Additional bands were also present between the ER and Golgi forms of the protein, indicating further intermediates that failed to form native disulfides. Hence, depletion of ERdj5 caused a dramatic attenuation of native disulfide formation and blocked trafficking of LDLR to the Golgi apparatus.

To further characterize the effect of ERdj5 depletion on LDLR folding, we cotransfected cells depleted of ERdj5 with LDLR and either wild-type, the H63Q mutant of ERdj5, or a mutant ERdj5 that had each active site mutated to AXXA (Figures 6E–6G). Each protein was expressed at similar levels, with each present at between 8× and 10× the level of endogenous ERdj5 prior to knockdown (Figure 6B). The ERdj5 constructs used have an altered codon bias to the endogenous gene and are not targeted by the shRNA. Coexpression of wild-type ERdj5 resulted in a reversal of the defect seen following shRNA depletion, with efficient formation of the correctly disulfide-bonded protein and trafficking to the Golgi (Figure 6E). However, cotransfection with the H63Q mutant of ERdj5 did not reverse the defect. In fact, a significant amount of the protein was present as high-molecular-weight aggregates that resided at the top of the separating gel (Figure 6F). In addition, the AXXA mutant of ERdj5 could not reverse the defect seen in LDLR folding, giving a very similar pattern of intermediates seen following knockdown (Figure 6G). These results demonstrate that the effect of ERdj5 depletion on LDLR folding is due to the absence of ERdj5 and that the disulfide exchange activity of ERdj5 is required to reverse the folding defect. Moreover, the reversal of the knockdown phenotype is dependent on the interaction of ERdj5 with BiP.

**Figure 7:**
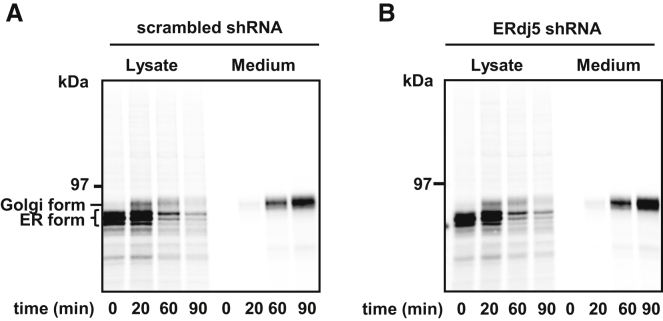
shRNA Knockdown of Endogenous ERdj5 Does Not Affect General ER Folding or Secretion (A and B) V5-tagged QSOX-1B was transiently transfected into HT1080 cells treated with scrambled shRNA (A) or ERdj5-specific shRNA (B). After 24 hr, cells were pulse labeled for 30 min and chased as indicated. Radiolabelled QSOX was immunoisolated from the cell lysate and medium with the V5 antibody and analyzed under reducing SDS-PAGE.

To determine whether the effect of ERdj5 on LDLR folding could be explained by a general defect in the trafficking of proteins following knockdown, we investigated the trafficking of a non-ERdj5 substrate. A V5-tagged version of human QSOX1B was used, as it is a soluble glycoprotein containing no structural disulfides (Alon et al., 2012) and becomes modified in the Golgi apparatus, allowing its trafficking and secretion to be monitored (Figure 7A). The ER form of the protein migrates as three distinct bands representing different glycoforms. The Golgi form of the protein becomes evident 20 min after the end of the pulse, which coincides with its appearance in the medium, indicating efficient trafficking and secretion. When the same experiment was carried out in ERdj5-knockdown cells, no effect was evident on the trafficking or secretion of QSOX1B (Figure 7B). These results demonstrate that ERdj5 knockdown does not cause a general defect in protein trafficking.

## Discussion

The substrate-trapping mutants of thioredoxin domain-containing proteins have been used successfully in the past to identify novel substrates for this family of enzymes (Jessop et al., 2007, 2009b; Zito et al., 2010). Here, we have extended these studies to determine the potential endogenous substrates for ERdj5. The isolation of proteins forming mixed disulfides with ERdj5 indicates that the enzyme is able to reduce either a pre-existing disulfide bond or a cysteine modified by sulfenylation or nitrosylation. Susceptible disulfides or modified cysteines are likely to be exposed at the protein surface to allow access by the enzyme. Mixed disulfides with ER-resident proteins known to contain solvent-accessible cysteines was not unexpected, though the formation of mixed disulfides with several PDI family members indicates that exchange of disulfides between these enzymes can occur. Since ERdj5 contains a thioredoxin domain with the lowest reduction potential of the PDI-family (Hagiwara et al., 2011), it might function to maintain the other PDIs in a reduced state, allowing them to participate in isomerization or reduction reactions. In support of such a hierarchy of disulfide exchange reactions is the fact that previous work with substrate-trapping mutants of PDI family members identified some mixed disulfides between PDIs, but never with ERdj5 (Jessop et al., 2009b). Hence, ERdj5 can reduce several other members of the family, but no other member can reduce ERdj5, despite it being present in the ER predominantly in an oxidized state (Riemer et al., 2009). How ERdj5 is itself reduced once its active site is oxidized remains unknown but may involve equilibration with the glutathione buffer in the ER lumen (Bulleid and Ellgaard, 2011).

Our results provide an indication of the breadth of endogenous proteins that can form mixed disulfides with ERdj5. The identification of these potential substrates is a crucial starting point to evaluate the function of this disulfide exchange protein in the biosynthesis of proteins entering the secretory pathway. As most of the previous work on this protein has focused on its role in degradation (Hagiwara et al., 2011; Ushioda et al., 2008), it is intriguing that our results demonstrate that its role is more extensive, being required for the efficient folding of the LDLR. The formation of the correct disulfides in this protein is likely to be a complex process requiring several different enzymes and ER chaperones. The specific role of ERdj5 in the folding of LDLR potentially is to reduce the non-native disulfides formed as an obligatory requirement for the correct folding of the protein (Jansens et al., 2002). This role is best exemplified during ERdj5 knockdown, which resulted in the perseverance of non-native disulfides in LDLR. As ERdj5 lacks the ability to isomerize disulfides (Ushioda et al., 2008), there would be a requirement for a second PDI family member to catalyze disulfide formation. It previously has been shown that ERp57, P5, and ERp46 also form mixed disulphides with LDLR (Jessop et al., 2009b), and it is highly likely that PDI itself is involved in catalyzing disulfide formation.

While the catalytic function of ERdj5 is as a reductase, we have shown that it can also associate noncovalently with its substrate. Crucially, the stable interaction between ERdj5 and LDLR was not dependent on the presence of the active-site, substrate-trapping mutation and was not abolished when BiP binding to the J domain was prevented. Indeed, the H63Q mutant of ERdj5 formed a more prolonged interaction with LDLR and increased ER residence. In addition, overexpression of ERdj5 caused a stabilization of the LDLR mutants, suggesting that binding of ERdj5 prevented entry into the ERAD pathway. These results strongly indicate that ERdj5 can act as a polypeptide-binding protein; its physical association with folding intermediates of LDLR may help to retain it in the ER, allowing correct disulfides and domain folding to occur. The ability of ERdj5 to bind to polypeptides is similar to a previously suggested role for PDI during the folding of procollagen (Wilson et al., 1998) and might be a general function of all the PDI family members.

As mentioned above, the polypeptide binding property of ERdj5 is not dependent on its ability to interact with BiP via its J domain. However, the reversal of the folding defect seen upon ERdj5 knockdown requires both its disulfide exchange activity and an interaction with BiP. Hence, the essential function of ERdj5 requires BiP binding via its J domain. One possible role for BiP in this regard would be to facilitate the release of ERdj5 from its substrate, as is the case for the release of ERdj3 from its substrate (Jin et al., 2008). The absence of BiP binding would cause prolonged ERdj5 binding and compromise its ability to act as a reductase or to allow other PDI family members access to LDLR to catalyze disulfide formation. Preventing BiP binding to ERdj5 also abolished the accelerated degradation of ERAD substrates upon overexpression of ERdj5 (Ushioda et al., 2008). Hence, for a productive role in both degradation and folding, ERdj5 needs to be able to bind to BiP via its J domain.

The identification of BiP as an interacting partner of ERdj5 binding via the J domain is entirely consistent with previous results (Cunnea et al., 2003). It recently has been shown that BiP itself may form a reversible disulfide bond that could influence its chaperone function (Wei et al., 2012). Therefore, an alternative explanation for our identification of BiP as a partner of the substrate-trapping mutant of ERdj5 that is released upon treatment with reducing agent could be more complex than simply due to BiP’s interaction with misfolded ERdj5 substrates. If BiP does form an internal intrachain disulfide to regulate its chaperone activity, then this disulfide may well be reduced by a PDI family member such as ERdj5. In support of such a role for ERdj5, we show here that the ERdj5 C/A mutant can form a mixed disulfide with BiP.

The reason for the presence of such a large family of oxidoreductases in the ER of mammalian cells has been speculated to be due to either substrate specificity or the ability to catalyze specific types of disulfide exchange (Hatahet and Ruddock, 2007). Both our previous results and those reported here demonstrate that there is not a clear demarcation between each enzyme in terms of their substrate specificity (Jessop et al., 2009b). ERdj5 is the only member of the PDI family so far whose function can be assigned to reduction of disulfides or reversibly modified cysteines. Such a reductase activity is required to ensure the correct folding of proteins entering the secretory pathway or in preparing misfolded proteins for dislocation to the cytosol for degradation.

## Experimental Procedures

### Expression Plasmids, Antibodies, and Inhibitors

A human ERdj5 DNA construct with CXXA mutation in the four thioredoxin homologous domains was synthesized by GenScript. A construct that included a V5 tag at the C terminus followed by a KDEL sequence was subcloned into pcDNA 3.1 (Invitrogen). The wild-type, AXXA, and H63Q mutants were generated using the appropriate primer pairs. The wild-type HA-tagged LDLR was from Guojun Bu (Mayo Clinic). V5-tagged QSOX1B was generated from QSOX1A constructed as described previously (Chakravarthi et al., 2007). The class 2 mutants G544V, P687L, and C646Y were generated from this construct using the appropriate primer pairs.

The commercially sourced antibodies used were mouse monoclonals (anti-HA [Sigma-Aldrich], anti-V5 [Invitrogen], anti-V5-conjugated agarose beads [Sigma-Aldrich], and anti-BiP [BD Transduction Laboratories]); rabbit monoclonals (anti-LDLR [C-terminal] and anti-UGGT1 [Epitomics]); and rabbit polyclonals (anti-actin [Sigma-Aldrich], anti-human α1AT [Dako], and anti-PrxIV [Abfrontier]). The Ero1α monoclonal antibody, 2G4, was from Roberto Sitia (San Raffaele Scientific Institute) (Ronzoni et al., 2010). The rabbit anti-ERdj5 was from Giannis Spyrou (Foundation for Biomedical Research, Academy of Athens) (Thomas and Spyrou, 2009). The rabbit anti-BiP antibody was from Richard Zimmerman (Universität des Saarlandes, Homburg) (Schäuble et al., 2012). Rabbit polyclonal antibodies for P5, PDI, and ERp57 have been described previously (Jessop and Bulleid, 2004).

### Cell Culture, Transfections, and shRNA Knockdown

HT1080 cells were maintained in Dulbecco’s modified Eagle’s medium (DMEM) supplemented with 10% fetal bovine serum (FBS). Cells were transfected with DNA using polyethylenimine (PEI) (Durocher et al., 2002). To create stable overexpression cells, transfected cells were placed on antibiotic (G418) selection for approximately 2 weeks until colonies appeared. For the shRNA-mediated knockdown, a human ERdj5-specific shRNA or a scrambled shRNA in pGFP-V-RS vector (OriGene) was transfected into subconfluent HT1080 cells. After 24 hr, shRNA-transfected cells were selected with 0.5 μg/ml puromycin for at least 5 days.

### Immunofluorescent Microscopy

Typically, HT1080 human fibroblast cells stably expressing ERdj5-V5 C/A mutant were permeabilized and fixed with methanol. Cells were labeled with a rabbit anti-PDI antibody and mouse anti-V5 antibodies, which were detected with the appropriate fluorescein isothiocyanate (FITC; Sigma-Aldrich) and Texas red (Abcam) secondary antibodies. Cells were imaged using a Zeiss Laser-Scanning Microscope (LSM) 5 Exciter.

### Mass Spectrometry

Confluent HT1080 cells and HT1080 cells stably overexpressing substrate-trapping ERdj5-V5 and the H63Q mutant were rinsed twice with PBS containing 20 mM *N*-Ethylmaleimide (NEM). Cells were lysed in 50 mM Tris-HCl buffer (pH 7.4) containing 1% (v/v) Triton X-100, 150 mM NaCl, 2 mM EDTA, and 0.5 mM phenylmethylsulfonyl fluoride (PMSF) supplemented with protease inhibitor cocktail (Roche). Clarified lysates were preincubated with protein A sepharose (Generon) before incubation with anti-V5-conjugated agarose beads (Sigma-Aldrich) for 16 hr at 4°C. Immunoisolates were washed three times with lysis buffer before incubation with 10 mM DTT for 5 min. The samples were centrifuged at 16,000 × *g* for 10 min to recover the eluted proteins, which were precipitated with 12% (w/v) trichloroacetic acid. Precipitated protein was solubilized in 25 mM ammonium bicarbonate and digested with trypsin.

Peptides were diluted 1:2 with 0.1% formic acid and separated using an UltiMate Nano LC (LC Packings) equipped with a PepMap C18 trap using a gradient of increasing acetonitrile concentration containing 0.1% formic acid. The eluate was sprayed into a QStar XL tandem mass spectrometer (AB SCIEX) and analyzed in the information-dependent acquisition mode; 1 s mass spectrometry (MS) followed by 3 s tandem mass spectrometry (MS/MS) was performed, analyzing the two most intense peaks seen by MS. These masses were then excluded from analysis for the next 60 s. MS/MS data for doubly and triply charged precursor ions were converted to centroid data, without smoothing, using the Analyst QS1.1 mascot.dll data import filter with default settings. The MS/MS data file generated was analyzed using the Mascot 2.1 search engine (Matrix Science) against the NCBInr database (February 2011; 12852469 sequences) with no species restriction. The data were searched with tolerances of 0.2 Da for the precursor and fragment ions, trypsin as the cleavage enzyme, one missed cleavage, NEM modification of cysteines as a fixed modification, and methionine oxidation selected as a variable modification. The Mascot search results were accepted if a protein hit included at least two peptides with ion scores above the homology threshold.

### Immunoisolation and Western Blots

Immunoisolation was performed by preclearing the cell lysates with protein A sepharose for 30 min, followed by incubation with the appropriate antibody and protein A sepharose for 16 hr at 4°C. Beads were washed three times in lysis buffer. Washed beads were heated at 95°C for 5 min in 200 mM Tris-HCl buffer (pH 6.8), 3% SDS, 10% glycerol, 1 mM EDTA, and 0.004% bromophenol blue in the presence (reducing) or absence (nonreducing) of 50 mM DTT prior to SDS-PAGE.

For western blotting, proteins were transferred to nitrocellulose membranes (LI-COR Biosciences), which were blocked in 5% (w/v) dried milk in 10 mM Tris-HCl buffer (pH 7.5) containing 150 mM NaCl and 0.1% (v/v) Tween 20 for 1 hr. Blots were incubated with primary antibody for 1 hr. LI-COR IRDye fluorescent secondary antibodies were used for detection at a 1:5,000 dilution. Blots were scanned using an Odyssey Sa Imaging System (LI-COR Biosciences).

### Metabolic Labeling and Pulse-Chase Analysis

HT1080 cells transfected with plasmids were incubated in medium lacking methionine and cysteine for 30 min and pulse labeled for 30 min with 11 μCi/ml of EXPRESS^35^S Protein Labeling Mix (PerkinElmer). The radiolabel was removed with two PBS washes. The cells were then incubated in complete medium to initiate the chase periods. To monitor the degradation of wild-type HA-LDLR and the class 2 mutants, transfected cells were incubated with or without MG132 (20 μM) in the medium during the starve, label, and chase periods.

Following the chase period, cells were washed twice with PBS supplemented with 20 mM NEM and lysed in lysis buffer containing 20 mM NEM. Postnuclear supernatant was obtained by centrifugation at 4°C, and immunoisolation was carried out as described above. SDS-PAGE gels were fixed, dried, and exposed to phosphorimager plate for between 24 and 72 hr. Radioactivity was detected using a Fujifilm FLA-7000 Phosphorimager. Quantification of band intensities was carried out using ImageJ software.

## Figures and Tables

**Table 1:** Mixed Disulfide Partners of ERdj5

Protein	Known Disulfides^a^ (Cysteines)	Coverage^b^ (%)	Present in H63Q
**ER-Resident Proteins**			

Peroxiredoxin-4	2	63	Y
BiP	(2)	61	Y
P5	2	54	Y
ERp72	3	46	Y
ERp57	3	45	Y
PDI	2	43	Y
Ero1	6	40	Y
Erp44	2	32	Y
UDP-glucose:glycoprotein glucosyltransferase	(13)	32	Y
ERp46	3	26	Y
Glucosidase II beta subunit	(17)	15	Y
Grp94	1	13	N
Formylglycine-generating enzyme	4	11	N
Hypoxia-upregulated protein 1 (Grp170)	(3)	8	Y
Lysyl hydroxylase isoform 2	(9)	3	Y

**Secreted Proteins**			

EGF-containing fibulin-like protein 1	15	39	Y
Laminin-5 beta3	27	37	N
Transforming growth factor-beta	5	30	N
Fibronectin	30	26	Y
Laminin subunit gamma	26	24	N
Collagen alpha-3(VI)	4	22	Y
Stanniocalcin-1	1	19	Y
Laminin B2	43	18	Y
Laminin B1	54	18	Y
Agrin	24	16	N
Granulins	5	12	N
Growth arrest-specific protein 6	16	8	N
Pentaxin	7	7	N
Transforming growth factor-beta binding protein	51	5	N
Collagen alpha-1(VI)	(19)	4	Y
Collagen alpha-2(IV)	6	3	N
Tenascin	42	2	N

**Non-ER Membrane Proteins**			

Low-density lipoprotein receptor (LDLR)	30	20	Y
Low-density lipoprotein receptor-related protein 8	27	12	Y
Death receptor 5	7	14	Y
Integrin beta-1 isoform 1A	28	12	Y
Amyloid-beta protein	(12)	5	N
MUC18 glycoprotein	5	4	N
NOTCH 2	115	4	N
Attractin-2	13	3	N
Mannose 6-phosphate receptor	2	2	Y
Epidermal growth factor receptor	25	2	N

a The number of disulfides that are known to form are as indicated. Where not known, the number of total cysteine residues in the protein is in parentheses.

b The value of percent coverage is for the C/A mutant of ERdj5.
